# Incidence of primary care chest pain consultations during the COVID-19 pandemic: an interrupted time series analysis with routine care data

**DOI:** 10.1186/s12875-024-02676-y

**Published:** 2024-12-21

**Authors:** Simone van den Bulk, Jasper W. A. van Egeraat, Annelieke H. J. Petrus, Mattijs E. Numans, Tobias N. Bonten

**Affiliations:** Department of Public Health and Primary Care, Leiden University Medical Center, Hippocratespad 21, Leiden, 2333 ZD The Netherlands

**Keywords:** Chest pain, COVID-19, Primary health care, Routinely collected health data, Interrupted time series analysis

## Abstract

**Background:**

The COVID-19 lockdown had profound effects on society and healthcare. Cardiology departments reported declines in chest pain evaluations and acute coronary syndrome (ACS) diagnoses. However, the pattern of chest pain in primary care is not clear yet. This study aims to assess the impact of the COVID-19 lockdown on the number of patients presenting with chest pain in primary care.

**Methods:**

Routine primary care data from the Extramural LUMC (Leiden University Medical Center) Academic Network (ELAN) in the Netherlands were used. Chest pain consultations from January 2017 to December 2020 were included. An interrupted time series analysis was performed to compare the incidence rate (IR) of chest pain consultations during the COVID-19 lockdown to the expected IR. Secondary outcomes were the type of consultations, referral proportions, and the IR of registered ACS diagnoses.

**Results:**

In total 9,908 chest pain consultations were included. During the COVID-19 lockdown the IR was 6.16 per 1000 person-years while the expected IR was 7.55 (95% CI 7.03–8.12). The immediate effect of the lockdown yielded an incidence rate ratio (IRR) of 0.62 (95% CI 0.50–0.77). A similar decrease was seen for ACS diagnoses (IRR 0.62, 95% CI 0.48–0.79), with no compensatory increase after the lockdown (IRR 1.04, 95% CI 0.89–1.21). Face-to-face consultations shifted to telephone consultations (*p* < 0.001) and hospital referrals decreased (9.9% vs. 19.0% (*p* < 0.001)).

**Conclusions:**

During the COVID-19 lockdown the number of chest pain consultations and registered ACS diagnoses in primary care decreased significantly. In addition, fewer patients were assessed face-to-face and fewer patients were referred to the hospital.

**Supplementary Information:**

The online version contains supplementary material available at 10.1186/s12875-024-02676-y.

## Key Message

- During the COVID-19 lockdown, significantly fewer patient consulted their general practitioner for chest pain.
- Fewer new diagnoses of ACS were registered by general practitioners during the COVID-19 lockdown.
- It remains unclear whether the number of ACS diagnoses truly decreased or if ACS cases were missed.

**Supplementary Information:**

The online version contains supplementary material available at 10.1186/s12875-024-02676-y.

## Introduction

During the COVID-19 lockdowns, a decline in new diagnoses of severe acute diseases other than COVID-19 was seen worldwide, including a 28–68% reduction in hospitalisation for patients with acute coronary syndrome (ACS) [1–3]. Emergency medical services (EMS) and hospitals in the Netherlands evaluated fewer patients with chest pain than before the lockdown [4, 5]. At the same time, an increased number of out-of-hospital cardiac arrests was reported, with higher mortality than before the pandemic [6].

Various hypotheses for the decline in ACS during these lockdowns have been proposed. Pathophysiological explanations have been offered concerning the decrease of known risk factors for ACS, such as diminished respiratory infections, particularly influenza, diminished air pollution from reduced traffic and industrial activity, less strenuous physical exercise and less stress [7–9]. However, the most commonly suggested explanations are behavioural, indicating missed ACS diagnoses due to patient or doctor delay [1, 5, 10–12].

In the Netherlands, the general practitioner (GP) serves as gatekeeper to secondary care. Typically, patients with chest pain initially contact their GP in 80% of cases, while 20% contact EMS directly [13]. This role became even more important during the pandemic, due to limited healthcare resources, presenting new dilemmas when deciding upon referral [14]. It is unknown if the reduction of chest pain consultations during lockdowns observed by EMS and hospitals, also occurred in primary care [15, 16]. Knowledge on this topic in the primary care setting is limited, with only one study which evaluated the number of myocardial infarction diagnoses and concluded that there was no reduction compared to previous years [10]. To the best of our knowledge, no study evaluated the presentation of patients with chest pain in primary care.

This study aims to evaluate the impact of the COVID-19 lockdown on the number of chest pain consultations in primary care. Secondary aims were to evaluate the impact of the COVID-19 lockdown on the number of ACS diagnoses and to compare the consultations types and referral rates for patients with chest pain before and during the lockdown.

## Methods

This study is reported following the RECORD guidelines for observational studies using routine care data [17].

### Study design and setting

A population-based cohort study was conducted using routine care data from primary care practices located in the greater Leiden and The Hague area in the Netherlands, affiliated with ELAN (Extramural Academic Network of the Leiden University Medical Centre) [18]. The ELAN data warehouse provides pseudonymised data from electronic health records, covering 440,000 patients at the start of the study period and 490,000 at the end. ELAN data used for this study comprises demographics, date and type of consultation, ICPC-coded diagnosis of the encounter and medical history (International Classification of Primary Care), ATC-coded medication prescriptions (Anatomic Therapeutic Chemical Classification System) and referrals to secondary care [19, 20]. In the Netherlands, GPs act as gatekeepers with practices opened from 8AM-5PM during which patients with chest pain symptoms can call assistants to assess the urgency of their symptoms, book an appointment or walk-in when experiencing chest pain symptoms. Patients with chest pain are assessed by history taking, physical examination and (if present in that GP practice) an ECG. No troponin tests are available on a regular basis. The criteria for referring patients are outlined in guidelines for Acute Coronary Syndrome of the Dutch College of General Practitioners (NHG) [21]. After referral of a patient with chest pain, the GP registers the hospital diagnosis based on discharge letters in the primary care electronic patient file. For this study, only primary care data was available.

The first COVID-19 lockdown in the Netherlands was initiated on March 16th 2020 (week 12) and measures were gradually lifted starting June 1st 2020 (week 23). Three time periods were defined: The COVID-19 lockdown period as March 14th to June 5th 2020. The pre-lockdown period as January 1st 2017 to March 13rd 2020 and the post-lockdown period as June 6th to December 31st 2020. We expected ACS diagnoses to increase again after the lockdown, because patients could have waited to contact health care with instable or increasing anginal complaints. Therefore, we also assessed the presence of a prolonged, compensatory effect, we also compared the post-lockdown period to the expected incidence rate post-lockdown.

### Study population

All patients registered in ELAN in 2017–2020 and aged 35 years and older were included. Consultations for chest pain were selected using ICPC-codes K01 (heart pain) and K02 (pressure/tightness of heart). Patients could have multiple consultations for chest pain. All contacts within eight weeks after the first consultation were considered follow-up. Consultations after this period were counted as a new episode of chest pain. Data from 2016 were used as a run-in period to secure incident ACS diagnoses at the start of the study period (January 2017). This run-in period of one year was concerned appropriate because patients with established heart disease visit their GP minimally twice per year for chronic cardiovascular risk disease management.

### Outcomes

The primary outcome was the weekly incidence rate (IR) of chest pain consultations during the COVID-19 lockdown, compared to the expected IR without lockdown. Secondary outcomes were the type of consultation for chest pain (face-to-face, telephone, home visit, e-consultation) and the proportion of referrals to emergency and cardiology departments during the lockdown period, compared to the pre-lockdown period. Patient characteristics during the lockdown period were compared to the pre-lockdown period. Patients who had a consultation before and during the lockdown were counted in both categories. Only the first consultation of each patient during each period was used for comparison.

Lastly, the observed IR of registered ACS diagnoses (defined as ICPC-codes K74 (ischaemic heart disease with angina) and K75 (acute myocardial infarction)) during the COVID-19 lockdown was compared to the expected IR without a lockdown.

A complete overview of the ICPC and ATC-codes used can be found in supplementary file 1.

### Statistical methods

Patient characteristics were analysed using descriptive statistics. Continuous variables are expressed as mean ± standard deviation (SD). Categorical data are presented as frequencies and percentages. T-test, chi-square statistics and Fisher’s exact test were used to compare outcomes where appropriate.

An interrupted time series analysis was performed to model the weekly number of consultations for chest pain during the full study period. We corrected for a generic trend by including a variable for weeks since the start of the study. The effect of the lockdown was estimated by including a variable set to one during lockdown and zero otherwise. To assess the trend in time during the lockdown, another variable was included, equal to the weeks since the start of the lockdown and zero before and after the lockdown. The same type of variables were included for the post-lockdown period. We corrected for seasonal effects by including harmonic functions up to the second order. The logarithm of the number of registered patients per week was included as offset to the model. The exact model specifications can be found in supplementary file 2. With our sample size, 80% power and a 5% significance level, the minimum detectable difference in the weekly incidence rate of chest pain between pre-lockdown and lockdown is a ± 14% change, corresponding to incidence rate ratios greater than 1.14 or smaller than 0.86 (see supplementary file 3).

Due to overdispersed data, a quasi-Poisson regression analysis was used to model the time series. We tested for autocorrelation using the Durbin Watson test with a maximum lag of up to 5 weeks. The effects of the included variables are presented as incidence rate ratios (IRR). All statistical analyses were performed using R version 4.3.2 and the Tidyverse collection of packages [22, 23]. P-values < 0.05 were considered significant. The statistical output is given in supplementary files 4 and 5.

## Results

In total 680 consultations for chest pain were recorded during the COVID-19 lockdown period and 9,228 during the pre-lockdown period. Patients consulting their GP for chest pain during lockdown had a mean age of 65 years (SD = 14) and 57% were women, comparable to patients presenting with chest pain before the lockdown (Table 1). Compared to before the lockdown, patients during lockdown more frequently had a history of cardiovascular disease (36% vs. 29%, p = < 0.001), diabetes mellitus (13% vs. 9.2%, *p* = 0.005) and hypertension (26% vs. 21%, *p* = 0.004) (Table 1). In line with these findings, patients presenting with chest pain during the lockdown used cardiovascular medication significantly more often: platelet inhibitors 32% vs. 23% (p = < 0.001), antihypertensives 56% vs. 48% (p = < 0.001), and statins 39% vs. 33% (*p* = 0.001). The proportion of patients diagnosed with ACS within 12 weeks after their consultation for chest pain was similar during and before the lockdown (9.9% vs. 12.0%, *p* = 0.11).

**Table 1:** Characteristics of patients consulting for chest pain before and during COVID-19 lockdown1

	Pre-lockdown period ^2^ *n* = 9,228	COVID-19 Lockdown *n* = 680	*p*-value^3^
Age (years (SD))^4^	65 (14)	66 (14)	0.140
Sex (male, n(%))	4,014 (43)	291 (43)	0.700
Medical history, n(%)			
History of CVD^5^	2,643 (29)	246 (36)	**< 0.001**
Coronary arterial disease	1,628 (18)	150 (22)	**0.004**
Cerebrovascular disease	484 (5.2)	50 (7.4)	**0.019**
Peripheral arterial disease	203 (2.2)	24 (3.5)	**0.025**
Diabetes mellitus	850 (9.2)	85 (13)	**0.005**
Hypertension	1,944 (21)	175 (26)	**0.004**
Hypercholesterolemia	527 (5.7)	41 (6.0)	0.700
COPD^6^	336 (3.6)	19 (2.8)	0.300
Depression	446 (4.8)	37 (5.4)	0.500
Medication, n(%)			
Antiplatelet therapy	2,166 (23)	218 (32)	**< 0.001**
Oral anticoagulation^7^	1,006 (11)	95 (14)	**0.014**
Antihypertensives	4,417 (48)	383 (56)	**< 0.001**
Beta-blockers	2,476 (27)	221 (33)	**0.001**
Statin	3,021 (33)	263 (39)	**0.001**
Insulin therapy	423 (4.6)	39 (5.7)	0.2
Type of contact, n(%)			
Live consultation	6,633 (72)	331 (49)	**< 0.001**
Telephone consultation	2,067 (22)	325 (48)	**< 0.001**
Home visit	523 (5.7)	22 (3.2)	**0.007**
E-consultation	5 (< 0.1)	2 (0.3)	0.078
Referred (yes), n(%)	1,787 (19)	67 (9.9)	**< 0.001**
Diagnosis of acute coronary syndrome^8^, n(%)	910 (9.9)	80 (12)	0.11

^1^Data are presented as mean ± sd for continuous outcomes and weighted proportion (%) for categorical outcomes; Wilcoxon rank sum test for continuous outcomes; Pearson’s Chi-squared test and Fisher’s exact test for categorical outcomes as applicable. ^2^ 2017 up to week 12 of 2020; ^3^P-values < 0.05 are considered statistically significant; ^4^Mean (SD); ^5^ CVD: cardiovascular disease, defined as: Myocardial infarction, coronary arterial disease, cerebrovascular accident, peripheral arterial disease; ^6^Chronic obstructive pulmonary disease; ^7^Vitamin K antagonists, DOAC (Direct Oral Anticoagulants); ^8^ Registered in electronic medical record within 12 weeks after consultation

### Incidence of chest pain consultations during the COVID-19 lockdown

**Fig. 1:**
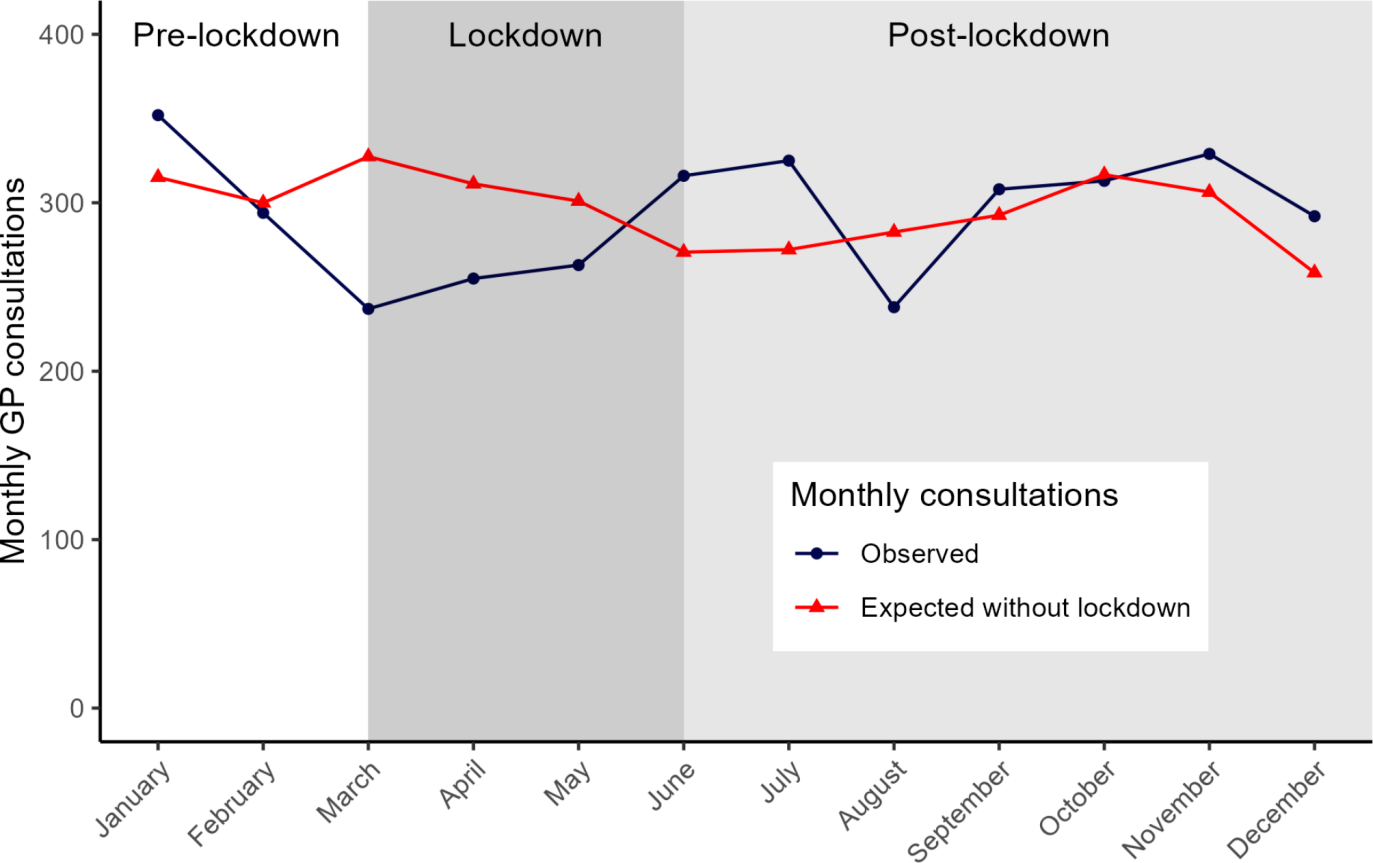
Monthly number of consultations for chest pain in primary care practices in 2020 with the COVID-19 lockdown (observed) and without the COVID-19 lockdown (expected). Expected consultations without the lockdown are based on data from January 2017 – December 2020

The IR for chest pain was 6.16 per 1000 person-years during lockdown. The expected IR in the absence of a lockdown was 7.55 per 1000 person-years (95% CI 7.03–8.12). After adjusting for seasonal effects and trend, the direct effect of the lockdown was an IRR of 0.62 (95% CI 0.50–0.77). After the lockdown, the observed number of consultations returned to expected levels (IRR 1.12, 95% CI 0.98–1.29) Fig. 1). No significant autocorrelation was detected (S*upplementary file 6)*.

### Type of consultation

The type of consultation for chest pain changed significantly during the COVID-19 lockdown. Consultations were less often face-to-face during lockdown compared to the pre-lockdown period, 72% vs. 49% (*p* < 0.001) and fewer home visits were made, 5.7% vs. 3.2% (*p* = 0.007). Telephone consultations increased from 22% before to 48% during lockdown (*p* < 0.001). E-consultations were hardly registered but increased during lockdown: five consultations (< 0.1%) before and two (0.3%) during lockdown (*p* = 0.078) (Table 1).

### Referrals to secondary care

GPs referred fewer patients with chest pain to the hospital during the COVID-19 lockdown compared to before the lockdown (9.9% vs. 19.0%, p = < 0.001).

### Incidence of ACS diagnoses during and after the COVID-19 lockdown

**Fig. 2:**
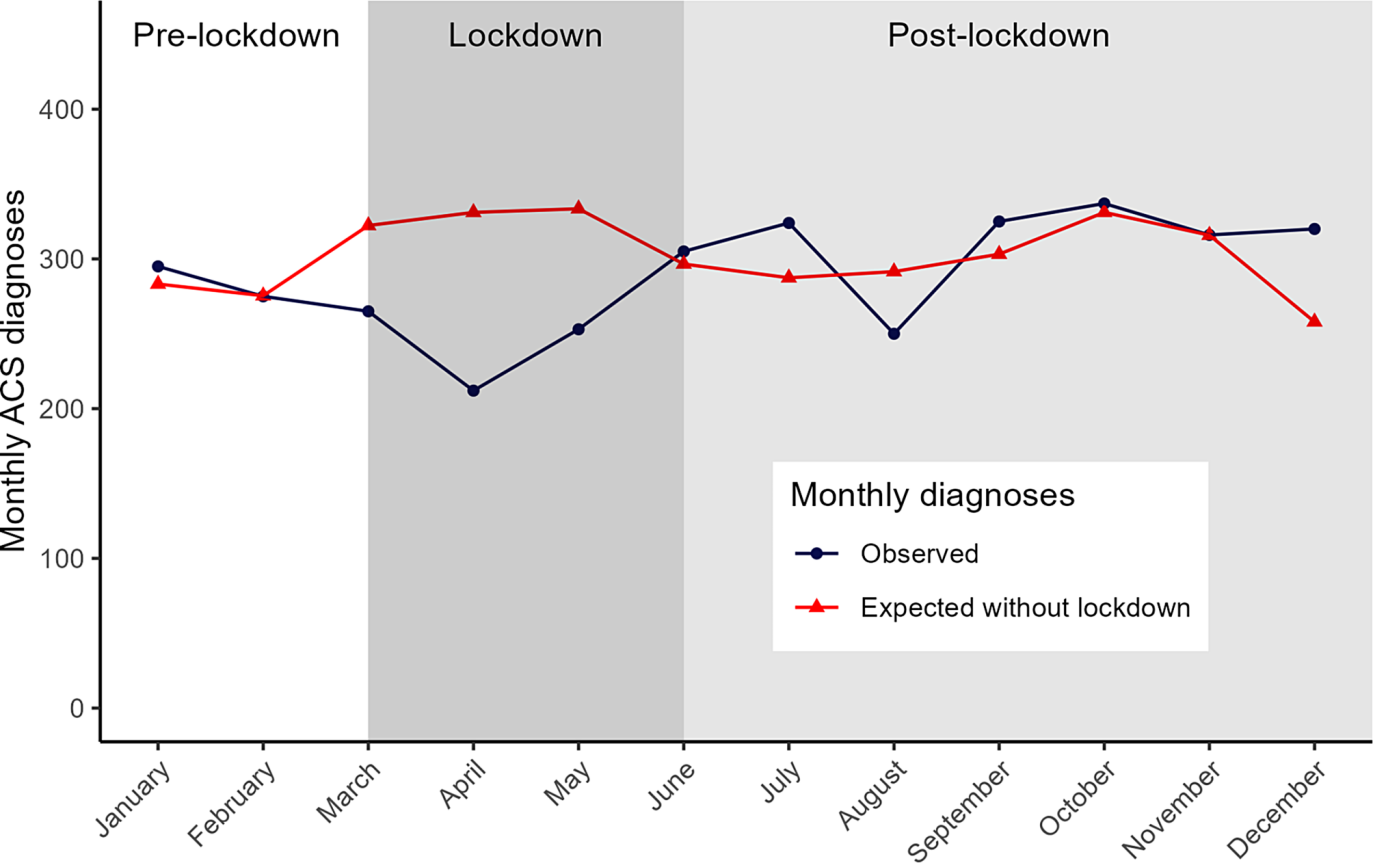
Monthly new registrations of acute coronary syndrome (ACS) diagnoses in primary care practices in 2020 with the COVID-19 lockdown (observed) and without the COVID-19 lockdown (expected). Expected consultations without the lockdown are based on data from January 2017 – December 2020

The IR for registered ACS diagnoses was 5.74 per 1000 person-years during the lockdown. The expected IR in the absence of a lockdown was 8.08 per 1000 person-years (95% CI 7.42–8.79). Before the lockdown, there was an increasing trend in the IR of ACS diagnoses. After adjusting for this trend and seasonal effects, the number of ACS diagnoses declined significantly at the start of the lockdown (IRR 0.62, 95% CI 0.48–0.79). Post-lockdown, the observed number of diagnoses resumed as expected (IRR 1.04, 95% CI 0.89–1.21) (Fig. 2).

## Discussion

This study shows a significant decline in primary care consultations for chest pain during the COVID-19 lockdown, returning to expected levels after the lockdown. Patients who consulted their GP for chest pain during lockdown were more likely to have cardiovascular disease or risk factors compared to patients with chest pain before the lockdown. Telephone consultations doubled during the lockdown, while face-to-face consultations and home visits decreased. Furthermore, GPs referred fewer patients with chest pain to the hospital during lockdown. The number of registered ACS diagnoses also decreased during the lockdown, with no compensatory increase after restrictions were lifted.

The decrease in patients presenting with chest pain in primary care during the COVID-19 lockdown and the decline in registered ACS diagnoses is in line with studies in secondary care [1, 2, 4]. During the lockdown, more patients had a history of cardiovascular disease or risk factors. Therefore, it seems that especially patients at low risk for cardiovascular disease refrained from contacting their GP for chest pain.

We found a significant decrease of registered ACS diagnoses in primary care during the COVID-19 lockdown, while Velek et al. [10] also evaluated the IR of cardiovascular disease in primary care during the lockdown and found no change for myocardial infarction. Differences in cohorts could explain these different results. For example, our cohort was approximately ten years older and we did not exclude patients with a history of ACS. Furthermore, our cohort is larger resulting in more power to detect statistical differences. However, it is also possible that regions within the Netherlands were affected differently by the lockdown.

The shift from mainly face-to-face contact to telephone consultations during lockdown corresponds with other studies in primary care [24, 25]. Surprisingly, we found few digital consultations, both before and during the lockdown. The increased use of telehealth during lockdown in primary and secondary care has been reported in many studies [24–26]. Our findings might be explained by the nature of the symptoms, that GPs and patients did not consider digital consultations appropriate to evaluate chest pain.

It still remains unclear whether the reduced number of observed ACS diagnoses reflects a true decrease of ACS or missed cases. To look for signs of missed ACS diagnoses in our data, we compared the post-lockdown IR of ACS with the expected IR. A rebound effect could indicate a part of missed ACS diagnoses during the lockdown period, because complications of the untreated disease would become apparent in the following months. No rebound effect was observed and missed ACS diagnoses were therefore not reflected in this study. Still, missed ACS diagnoses may also present as heart failure, arrhythmias or cardiac arrest after a longer time period, which we could not assess in our dataset.

It is important to note that our study population was defined by consultations for chest pain and ACS, coded as such by the GP at the end of the consultation. There are other codes a GP can use when suspecting a specific aetiology for the pain. For example, a patient with chest pain, which the GP interpreted as a COVID-19 related symptom and coded as COVID-19 or respiratory disease. This could partially explain the reduced number of observed chest pain consultations and it is conceivable that ACS diagnoses in this group were missed because the chest pain was misinterpreted. In the present study, we were unable to evaluate patients coded differently by the GP.

### Strengths and limitations

Interrupted time series analysis offers the possibility to evaluate healthcare changes caused by unique, unexpected historical circumstances. In unforeseen situations, like the COVID-19 pandemic, a prospective study design is not possible. Interrupted time series is a strong quasi-experimental study design and a valid alternative [27, 28]. However, our study is observational in nature, which prevents drawing causal conclusions. Other factors than the lockdown period might have contributed to the observed decline in chest pain and ACS diagnoses. Still, by using routine care data, we were able to include a large number of patients and look for trends over a longer time period. A limitation of routine care data is that the data were not collected for research purposes and the quality depends on the registration accuracy of GPs. For example, there is a risk of misclassification bias since GPs could have used other ICPC codes for chest pain (for example L04: pain attributed to chest wall). Also, misclassification could occur when GPs do not register the final hospital diagnosis correctly in their primary care electronic patient file. This may lead to underestimation of the disease prevalence. However, we do not have reason to assume that the magnitude of this misclassification changed over the years. As such, these forms of misclassification will not affect our primary endpoint (comparison of consultation rates over time).

### Implications

The cause of the decline in ACS diagnoses remains unclear. One possibility is a true reduction in the incidence of ACS by the lockdown or the COVID-19 pandemic as a whole. Proposed explanations are environmental- and lifestyle changes that may reduce the risk of plaque erosion [7, 8, 10, 11]. Additionally, patients at high risk for ACS may have died from COVID-19 first due to the shared risk factors for ACS and severe COVID-19 infections (competing risks). Alternatively, ACS diagnoses may have been missed due to patients avoiding care due to fear of infection with COVID-19 in healthcare settings, misjudgement of the urgency of symptoms, assumptions of insufficient healthcare capacity, or solidarity with healthcare providers to alleviate pressure on the healthcare system with assumed non-COVID-19 related complaints [11]. Lastly, health care professionals might have missed ACS diagnoses due to altered work conditions, including fewer face-to-face consultations and more remote (i.e. telephone and digital) consultations [25]. These conditions may have made interpreting chest pain symptoms and assessing the urgency more challenging. The scarcity of healthcare resources also presented new dilemmas when deciding upon referral [14].

## Conclusion

Consultations for chest pain and registered ACS diagnoses in primary care decreased significantly during the COVID-19 lockdown. Consultations for chest pain were less often face-to-face and hospital referrals decreased. The cause of the decline in ACS remains unclear, however this study found no indication of missed ACS.

## Electronic supplementary material

Below is the link to the electronic supplementary material.

Supplementary Material 1

## Data Availability

The data that support the findings of this study are available from ELAN (https://elanresearch.nl), but restrictions apply to the their availability. These data were used under license for the current study and thus are not publicly available. Data are, however, available from the authors upon reasonable request and with permission of ELAN.

## Abbreviations

ACS: Acute coronary syndrome
ATC: Anatomic therapeutic chemical classification system
CI: Confidence interval
COPD: Chronic obstructive pulmonary disease
CVD: Cardiovascular disease
ECG: Electrocardiogram
ELAN: Extramural Academic Network of the Leiden University Medical Centre
EMS: Emergency medical services
GP: General practitioner
ICPC: International classification of primary care
IR: Incidence rate
IRR: Incidence rate ratio
SD: Standard deviation
