# Supplementary material for: Incidence of primary care chest pain consultations during the COVID-19 pandemic: an interrupted time series analysis with routine care data
Source: BMC Prim Care. 2024 Dec 21;25:433. doi: 10.1186/s12875-024-02676-y (PMC11662427; doi:10.1186/s12875-024-02676-y)
Supplement: Supplementary file 1 — Supplementary Material 1 [file 12875_2024_2676_MOESM1_ESM.docx]

Supplementary data

# **Supplementary file 1 – used ATC and ICPC codes**

**International Classification of Primary Care (ICPC) codes [1]**

Selection of patients

- K01 Heart pain
- K02 Pressure/tightness of heart

Medical history

History of cardiovascular disease

- K74 Ischaemic heart disease w. angina
- K75 Acute myocardial infarction
- K76 Ischaemic heart disease w/o angina
- K77 Heart failure
- K89 Transient cerebral ischaemia
- K90 Stroke/cerebrovascular accident
- K92 Atherosclerosis/PVD

Coronary artery disease

- K74 Ischaemic heart disease w. angina
- K75 Acute myocardial infarction

Cerebrovascular disease

- K89 Transient cerebral ischaemia
- K90 Stroke/cerebrovascular accident

Peripheral artery disease

- K92 Atherosclerosis/PVD

Diabetes mellitus

- T89 Diabetes insulin dependent
- T90 Diabetes non-insulin dependent

Hypertension

- K86 Hypertension uncomplicated
- K87 Hypertension complicated

Hypercholesterolemia

- T93 Lipid disorder

Chronic obstructive pulmonary disease

- R95 Chronic obstructive pulmonary disease

Depression

- P76 Depressive disorder

Diagnosis of acute coronary syndrome

- K74 Ischaemic heart disease w. angina
- K75 Acute myocardial infarction

**Anatomic, Therapeutic, Chemical Classification System (ATC) codes [2]**

Medication

Antiplatelet therapy

- B01AC Platelet aggregation inhibitors excl. heparin

Oral anticoagulation

- B01AA Vitamin K antagonists
- B01AE Direct thrombin inhibitors
- B01AF Direct factor Xa inhibitors

Antihypertensives

- C02 Antihypertensives
- C07 Beta blocking agents
- C08 Calcium channel blockers
- C09 Agents acting on the renin-angiotensin system

Beta-blockers

- C07 Beta blocking agents

Statin

- C10 Lipid modifying agents

Insulin therapy

- A10A Insulin and analogues

1. Organization, W.H. *International Classification of Primary Care, 2nd edition (ICPC-2)*. 2015; Available from: <https://www.who.int/standards/classifications/other-classifications/international-classification-of-primary-care>.

2. Methodology, W.C.C.f.D.S. *ATC classification index with DDDs.* 2021; Available from: <https://www.whocc.no/atc_ddd_index/>.

# **Supplementary file 2 – Analytical model**

Model formula:

$$\log(y_{i})=\beta_{0}+\beta_{1}T_{i}+\beta_{2}R_{i}+\beta_{3}R_{i}*T_{i}+\beta_{4}A_{i}+\beta_{5}A_{i}*T_{i}+\beta_{6}\text{sin1}+\beta_{7}\text{sin2}+\beta_{8}\text{cos1}+\beta_{9}\text{cos2}+\log(n_{i})$$

- y_i: The count of GP consultations in week i
- T_i: Number of weeks since the start of the study, where the first week is defined as week 0 (2017-01-01 to 2017-01-07)
- R_i: indicator which is 0 in the prelockdown period, 1 in the lockdown, and 0 again after. Specifically:
  - R_i = 0 for 0 <= i <= 166,
  - R_i = 1 for 167 <= i <= 178,
  - R_i = 0 for i >= 179.
- A_i: indicator which is 1 in the post lockdown period, and 0 everywhere else
  - A_i = 0 for i <= 178
  - A_i = 1 for i >= 179
- For the harmonic functions, d_i is the amount of days between the first day of week i and the first day of the corresponding year:
  - sin1: $\sin(2\pi d_{i}/365.25)$
  - cos1: $\cos(2\pi d_{i}/365.25)$
  - sin2: $\sin(4\pi d_{i}/365.25)$
  - cos2: $\cos(4\pi d_{i}/365.25)$
- n_i: The number of enrolled patients in GP database in week i

lockdown start = 2020-03-14 lockdown end = 2020-06-05

# **Supplementary file 3 – Minimum detectable difference**

To determine the minimum detectable difference in weekly incidence rate between pre lockdown and lockdown we performed a simulation. We have 167 weeks of pre lockdown, 12 weeks of lockdown, and 30 weeks of post-lockdown. For this simulation we assume no difference between pre- and post-lockdown, no trends and an overdispersion of 2.

In the Netherlands, the incidence rate for chest discomfort was 8.1 per 1000 patient years in 2019 according to rates published previously [1]. We modelled the seasonal effect with a cosine wave of one year period, changing the incidence rate by +15% during winter and -15% during summer, based on seasonal effects on acute coronary syndromes reported previously [2].

The weekly number of consultations was generated with a quasi-Poisson distribution with a mean of

$\lambda_{i}=\text{IR}\cdot\text{PY}_{i}\left( 1+0.15\cos\left( \frac{2\pi i}{52} \right)+x\cdot\text{LD}_{i} \right),$ where

IR is the incidence rate (modelled to be 8.1/1000 patient years),

$\text{PY}_{i}$ the number of patient years in week i,

$0.15\cos\left( \frac{2\pi i}{52} \right)$ represents the seasonal effect of +15% during winter and -15% during summer,

x the simulated effect of the lockdown, and

$\text{LD}_{i}$ an indicator that is 1 during the 12 lockdown weeks and 0 otherwise.

Based on a significance level of 5% and a power of 80%, the minimum detectable difference is given by the smallest x for which a quasi Poisson regression on this simulated dataset detects a significant coefficient for the lockdown indicator 80% of the times.

By generating data and fitting the model 1000 times for varying values of x, we find a minimum detectable difference of ±0.14. Thus, incidence rate ratios greater than 1.14 and smaller than 0.86 are detectable.

1. Cijfers Ziekten op jaarbasis in Nederland - incidentie en prevalentie | Nivel. https://www.nivel.nl/nl/zorg-en-ziekte-in-cijfers/cijfers-ziekten-op-jaarbasis. Accessed 19 Nov 2024.

2. Li Y, Du T, Lewin MR, Wang H, Ji X, Zhang Y, et al. The seasonality of acute coronary syndrome and its relations with climatic parameters. Am J Emerg Med. 2011;29:768–74.

# **Supplementary file 4 – Output chest pain consultations**

| **Variable** | **Estimate** | **Std Error** | **P-value** |
| --- | --- | --- | --- |
| Intercept | -8.74 | 0.024 | < 2e-16 |
| t | <-0.001 | <0.001 | 0.010 |
| in_lockdown | -0.474 | 0.111 | 2.92e-05 |
| t_lockdown | 0.045 | 0.015 | 0.003 |
| post_lockdown | 0.115 | 0.071 | 0.105 |
| t_post_lockdown | -0.006 | 0.004 | 0.142 |
| sin1 | 0.001 | 0.017 | 0.955 |
| sin2 | -0.013 | 0.016 | 0.433 |
| cos1 | 0.068 | 0.017 | <0.001 |
| cos2 | -0.047 | 0.016 | 0.004 |

| **Characteristic** | **IRR***^1^* | **95% CI***^1^* | **p-value** |
| --- | --- | --- | --- |
| t | 1.00 | 1.00, 1.00 | 0.010 |
| in_lockdown | 0.62 | 0.50, 0.77 | <0.001 |
| t_lockdown | 1.05 | 1.02, 1.08 | 0.003 |
| post_lockdown | 1.12 | 0.98, 1.29 | 0.10 |
| t_post_lockdown | 0.99 | 0.99, 1.00 | 0.14 |
| sin1 | 1.00 | 0.97, 1.03 | >0.9 |
| sin2 | 0.99 | 0.96, 1.02 | 0.4 |
| cos1 | 1.07 | 1.03, 1.11 | <0.001 |
| cos2 | 0.95 | 0.93, 0.98 | 0.004 |
| *^1^*IRR = Incidence Rate Ratio, CI = Confidence Interval | | | |

Residual plot: (x-axis: predicted value, y- axis: residual (observed value – predicted value)


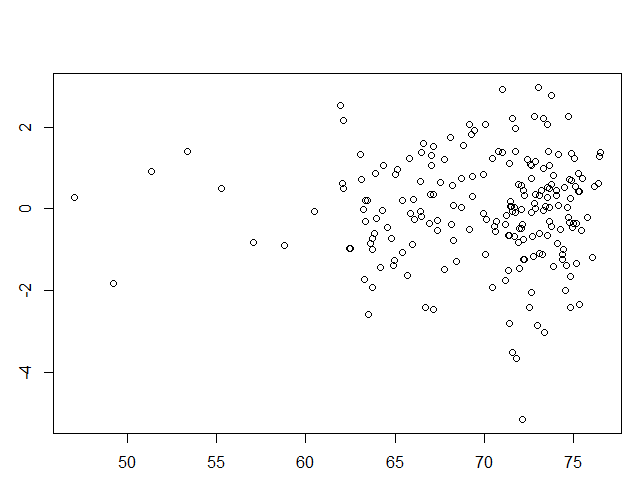


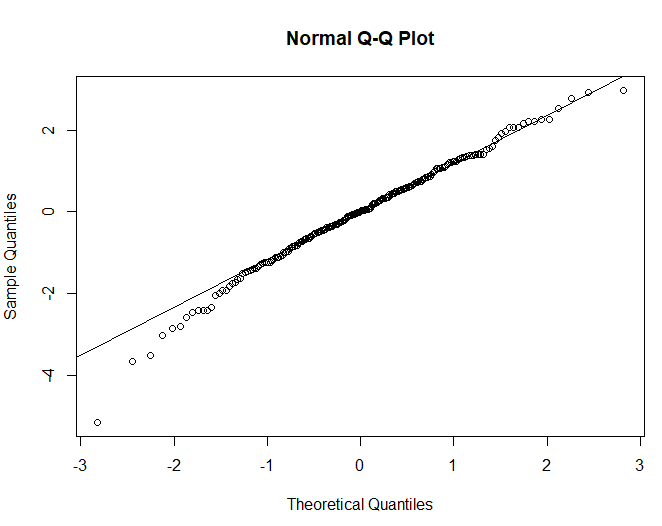


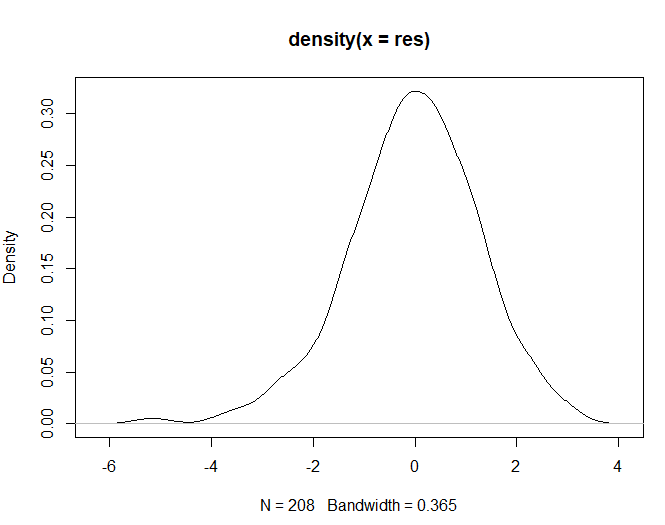


Residual vs date


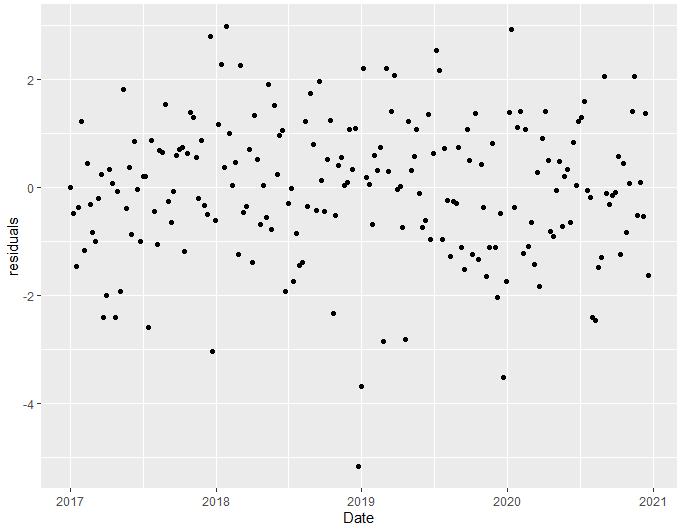


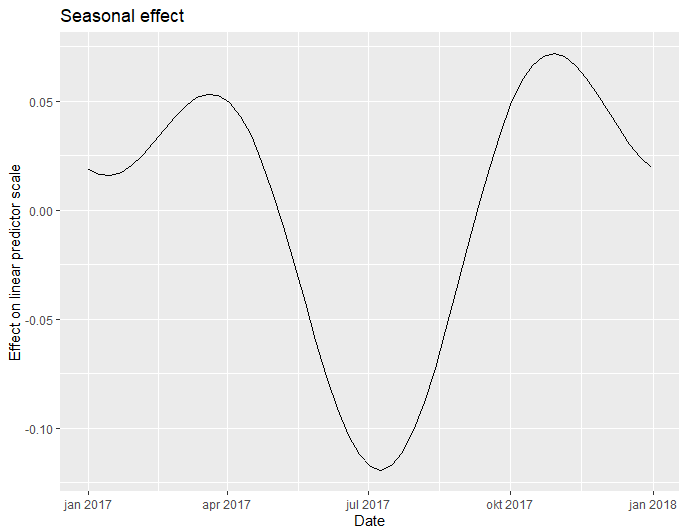


# **Supplementary file 5 – Output acute coronary syndrome diagnoses**

| **Variable** | **Estimate** | **Std Error** | **P-value** |
| --- | --- | --- | --- |
| Intercept | -8.987 | 0.030 | <2e-16 |
| t | 0.001 | <0.001 | 0.006 |
| in_lockdown | -0.476 | 0.127 | <0.001 |
| t_lockdown | 0.022 | 0.017 | 0.202 |
| post_lockdown | 0.039 | 0.080 | 0.627 |
| t_post_lockdown | <-0.001 | 0.004 | 0.976 |
| sin1 | 0.017 | 0.020 | 0.395 |
| sin2 | -0.050 | 0.0187 | 0.009 |
| cos1 | 0.002 | 0.020 | 0.939 |
| cos2 | -0.066 | 0.019 | 0.001 |

| **Characteristic** | **IRR***^1^* | **95% CI***^1^* | **p-value** |
| --- | --- | --- | --- |
| t | 1.00 | 1.00, 1.00 | 0.006 |
| in_lockdown | 0.62 | 0.48, 0.79 | <0.001 |
| t_lockdown | 1.02 | 0.99, 1.06 | 0.2 |
| post_lockdown | 1.04 | 0.89, 1.21 | 0.6 |
| t_post_lockdown | 1.00 | 0.99, 1.01 | >0.9 |
| sin1 | 1.02 | 0.98, 1.06 | 0.4 |
| sin2 | 0.95 | 0.92, 0.99 | 0.009 |
| cos1 | 1.00 | 0.96, 1.04 | >0.9 |
| cos2 | 0.94 | 0.90, 0.97 | <0.001 |
| *^1^*IRR = Incidence Rate Ratio, CI = Confidence Interval | | | |

# **Supplementary file 6 – Results of the Durbin Watson test**

Chest pain consultations

| **Lag (weeks)** | **Autocorrelation** | **DW statistic** | **p-value** |
| --- | --- | --- | --- |
| 1 | 0.017 | 1.958 | 0.938 |
| 2 | -0.004 | 1.995 | 0.694 |
| 3 | 0.040 | 1.899 | 0.804 |
| 4 | -0.061 | 2.101 | 0.194 |
| 5 | -0.059 | 2.093 | 0.268 |

ACS diagnoses

| **Lag (weeks)** | **Autocorrelation** | **DW statistic** | **p-value** |
| --- | --- | --- | --- |
| 1 | 0.089 | 1.804 | 0.238 |
| 2 | -0.034 | 2.044 | 0.584 |
| 3 | -0.066 | 2.094 | 0.356 |
| 4 | -0.094 | 2.144 | 0.198 |
| 5 | 0.020 | 1.894 | 0.562 |
